# RETRACTION: Efficacy and Safety of Oral Probiotic Supplementation in Mitigating Postoperative Surgical Site Infections in Patients Undergoing Colorectal Cancer Surgery: A Systematic Review and Meta‐Analysis

**DOI:** 10.1111/iwj.70575

**Published:** 2025-04-18

## Abstract

**RETRACTION:** ChenJ. , ZhaoJ. , WuH. , WangT. , and GaoC. , “Efficacy and Safety of Oral Probiotic Supplementation in Mitigating Postoperative Surgical Site Infections in Patients Undergoing Colorectal Cancer Surgery: A Systematic Review and Meta‐Analysis,” International Wound Journal 21, no. 4 (2024): e14603, 10.1111/iwj.14603.38155392 PMC10961893

The above article, published online on 28 December 2023, in Wiley Online Library (http://onlinelibrary.wiley.com/), has been retracted by agreement between the journal Editor in Chief, Professor Keith Harding; and John Wiley & Sons Ltd. Following an investigation by the publisher, all parties have concluded that this article was accepted solely on the basis of a compromised peer review process. The editors have therefore decided to retract the article. The authors did not respond to our notice regarding the retraction.

**RETRACTION:** J. Chen , J. Zhao , H. Wu , T. Wang , and C. Gao , “Efficacy and Safety of Oral Probiotic Supplementation in Mitigating Postoperative Surgical Site Infections in Patients Undergoing Colorectal Cancer Surgery: A Systematic Review and Meta‐Analysis,” International Wound Journal 21, no. 4 (2024): e14603, 10.1111/iwj.14603.38155392 PMC10961893

The above article, published online on 28 December 2023, in Wiley Online Library (http://onlinelibrary.wiley.com/), has been retracted by agreement between the journal Editor in Chief, Professor Keith Harding; and John Wiley & Sons Ltd. Following an investigation by the publisher, all parties have concluded that this article was accepted solely on the basis of a compromised peer review process. The editors have therefore decided to retract the article. The authors did not respond to our notice regarding the retraction.

